# *α*-Fluctuating Nakagami-*m* Fading Model for Wireless Communications

**DOI:** 10.3390/s25113430

**Published:** 2025-05-29

**Authors:** Aleksey S. Gvozdarev

**Affiliations:** Department of Intelligent Radiophysical Information Systems (IRIS), Physics Faculty, P.G. Demidov Yaroslavl State University, Yaroslavl 150003, Russia; asg.rus@gmail.com

**Keywords:** fading channel, statistical description, Fluctuating Nakagami, error rate, outage, capacity, amount of fading, diversity gain, 89.70.+c, 84.40.Ua, 94A15, 94A40

## Abstract

This research introduces and studies the performance of the α-Fluctuating Nakagami-*m* model, which addresses the limitations of conventional models for wireless communications. For the assumed channel model, the research presents a complete first-order statistical description (including the probability density function (PDF), cumulative distribution function (CDF), moment generating function (MGF), and raw moments) and provides closed-form results for system performance (assessed in terms of outage probability, average bit error rate (ABER), and channel capacity). All of the expressions have the same numerical complexity as the base-line Fluctuating Nakagami-*m* model, and are accompanied by their high signal-to-noise ratio (SNR) asymptotics. The derived results helped to identify the amount of fading (AoF) and diversity/coding gain of the proposed channel model. In-depth analysis of the system performance was carried out for all possible fading channel parameter values. Numerical analysis of the proposed solutions demonstrated their high computational efficiency. The comparison with experimental results demonstrated that the model offers enhanced flexibility and better characterization of fading regimes. Numerical analysis and simulation results show a high degree of correspondence with the analytical work and help study the dependence of channel nonlinearity effects on overall system performance.

## 1. Introduction

The design of modern wireless communication systems and wireless sensor networks (WSNs) that operate in the presence of multipath fading channels relies heavily on the ability to adapt system parameters based on the overall performance assessment [1,2,3,4,5]. Thus, it is important for researchers and engineers to have at hand concise channel models that are valid for various propagation conditions and verified through real-life measurements. The main drawback of existing models lies in the complexity of their mathematical description, especially when effects such as fading and shadowing are taken into account (see, for example, [6]). As a result, much effort has been devoted to the development of adequate simplified models, ranging from the pioneering works [7,8,9,10,11,12,13,14] to the most recent ones [15,16,17,18]. One of the main aspects of the majority of these models is how they convey shadowing. It is broadly accepted that such effects can be incorporated via Gamma [10], Inverse-Gamma, or Nakagami [7] distributions. These approaches have a solid foundation (both analytical and experimental [19]), although they lead to very complex results regarding overall system performance (e.g., results expressed in terms of multivariate hypergeometric, multivariate Meijer G-, or Fox H-functions [20,21,22,23,24]). Recently, it was demonstrated that this issue can be circumvented by assuming a uniform distribution for shadowing of the multipath components [18]. Such an approach not only allows closed-form derivations of reasonable complexity but also makes it possible to account for heavy-tailed fading, which is valuable since it reflects poor propagation conditions.

From a practical viewpoint, this model can be further improved—gaining new scope and flexibility—by introducing additional degrees of freedom (i.e., accounting for more effects). This can be achieved by assuming possible nonlinear wireless signal transformations, leading to the so-called α-variate models. First introduced in [25], this approach has since gained sufficient traction by demonstrating its wide applicability in various practical scenarios [17,26,27,28,29,30,31]. It has led to several forms of α-variate models (see, for example, [6,17,26,29,32,33,34,35,36,37,38,39,40,41]).

The introduction of the α parameter is typically motivated by the need for greater flexibility in characterizing real-world wireless propagation environments. Traditional fading models, including Nakagami-*m* and its extensions, have been successful in modeling a wide range of communication scenarios. However, empirical studies have repeatedly shown that standard fading distributions often fail to fully capture the statistical variations observed in practice. The α parameter, first introduced in the α-μ fading model by M. D. Yacoub [25], has been instrumental in accounting for nonlinearities and power-law characteristics in wireless channels—phenomena that classical models do not sufficiently represent.

In wireless communications, propagation can rarely be assumed to be homogeneous [42,43]. The presence of obstacles, scatterers, and varying atmospheric conditions introduces fluctuations that cause deviations from conventional Gaussian assumptions (which stem from the central limit theorem applied to multipath components). The aforementioned Fluctuating Nakagami-*m* model already provides an improvement over classical models by incorporating both line-of-sight (LoS) and non-line-of-sight (NLoS) conditions with a fluctuating power component. However, the introduction of the α parameter further enhances this model by accounting for additional irregularities in the propagation environment. Specifically, it offers a more accurate representation of small-scale fading in complex scenarios.

The α parameter also plays an important role (as verified experimentally) in modeling modern wireless technologies (i.e., fitting the distribution of channel coefficients), including Device-to-Device (D2D) communication [36], underwater acoustic communication systems [36], cellular networks [17], indoor LoS and NLoS mm-Wave communications [44], THz communication for 6G [45], and more. In these contexts, the fading characteristics exhibit complex spatial and temporal variations that are better captured by the enhanced flexibility offered by α.

Moreover, empirical data from various environments—such as urban microcells, vehicular communication systems, and D2D setups—have shown that fading distributions often exhibit heavy-tailed behaviors or non-Gaussian fluctuations. The α parameter enables a more flexible characterization of these conditions [17], allowing the Fluctuating Nakagami-*m* (FN) model to be modified to better match measured data. Furthermore, the α modification provides greater control over the severity of fading, which is crucial for optimizing wireless network performance [17,40]. This flexibility proves useful for evaluating key system performance metrics, including bit error rate (BER), outage probability, and channel capacity.

Thus, to extend the scope of the Fluctuating Nakagami-*m* model [18], this research presents and studies its α-transformed variant, i.e., the α-Fluctuating Nakagami-*m* (α-FN) model.

It should be specifically noted that the proposed α-FN model, in addition to improving analytical tractability, maintains the mathematical structure and complexity level of the baseline model [18]. By incorporating an additional degree of freedom, it enables the derivation of more generalized expressions for performance metrics, thus facilitating efficient system design and optimization. The model unifies various existing distributions within a single mathematical framework. This unification is particularly valuable for designing adaptive communication systems that must operate across diverse propagation conditions.

Additionally, the inclusion of α helps bridge the gap between small-scale fading and shadowing effects. Many real-world environments exhibit composite fading, where large-scale shadowing overlaps with small-scale multipath fading. The α-FN model can more accurately characterize such scenarios, leading to improved precision in system performance prediction. This is especially relevant in satellite and airborne communication systems, where signals traverse multiple atmospheric layers with varying densities and turbulence levels.

The necessity of introducing the α modification to the Fluctuating Nakagami-*m* model is justified by its ability to generalize existing models, enhance analytical tractability, improve empirical data fitting, and extend applicability to both modern and future wireless communication systems. By incorporating this additional parameter, researchers and engineers are equipped with a more versatile tool for modeling, analyzing, and optimizing wireless networks under diverse and challenging propagation conditions.

The major contributions of this research can be summarized as follows:

- A closed-form probabilistic description of the α-Fluctuating Nakagami-*m* fading channel model suitable for WSN channel description is introduced, and its first-order statistical characteristics of the instantaneous signal-to-noise ratio (SNR) (i.e., probability density function (PDF), cumulative distribution function (CDF), moment generating function (MGF) and raw moments) are derived.
- Exact expressions for the high-SNR asymptotics are derived for all the assumed channel characteristics.
- A broad comparison of the proposed α-Fluctuating Nakagami-*m* model with the most widely used model in wireless communications is performed. The results obtained revealed its ability to account for both heavy and light fading in terms of the specific channel parameters.
- The exact expressions are derived for *(a)* amount of fading (AoF); *(b)* outage probability (OP); *(c)* average bit error rate (ABER) for both coherent and non-coherent modulations; *(d)* ergodic channel capacity (EC).
- All evaluated channel performance metrics are appended with the closed-form expressions for their high-SNR asymptotics.
- The derived expressions helped to evaluate the amount of fading (AoF) and the diversity/coding gain of the channel.
- The practical validation of the applicability of the α-Fluctuating Nakagami-*m* model for wireless communications was performed on a set of D2D experimental data.
- A numerical study of the derived expressions demonstrated their high computational efficiency, that is, high computational speedup and low relative error compared to brute-force numerical integration.
- An intensive analysis of the joint dependence of all the derived channel performance metrics on the parameters of the α-Fluctuating Nakagami-*m* is performed for various fading scenarios (including strong and light fading).

The remainder of the article is organized as follows. Section 2 presents a brief introduction into the underlying Fluctuating Nakagami-*m* model; Section 3 presents the physical model for the proposed α-Fluctuating Nakagami-*m* channel and derives its statistical representation (in terms of PDF, CDF, and MGF), as well as their high-SNR asymptotics, raw moments, and AoF; Section 4 presents the derived results for the specific application of the α-Fluctuating Nakagami-*m* model in wireless fading communications (that is, the closed-form expressions for outage probability, average bit error rate, and ergodic capacity); Section 5 presents the results of an in-depth numerical analysis of the evaluated expressions for all specific channel and system parameter values; and concluding remarks are drawn in Section 6.

## 2. Fluctuating Nakagami Fading Channel Model

The fluctuating Nakagami-m fading distribution was initially proposed in [18] as the ratio of two independent random variables: the Nakagami-distributed and power-transformed uniformly distributed one, with the channel coefficient envelope *R* given by

(1) $$R=\sqrt{\sum_{i=1}^{m}U^{-\frac{1}{m_{s}}}X_{i}^{2}+\sum_{i=1}^{m}U^{-\frac{1}{m_{s}}}Y_{i}^{2}}.$$

It is assumed that the complex random variable $X_{i}+i\;Y_{i}$ (signal in each of the multipath clusters) follows the zero mean circular symmetric complex normal distributions (i.e., $X_{i}+i\;Y_{i}∼\mathrm{CN}\left(0,2\sigma^{2}\right)$) with *m* being a total number of such clusters. Each component is assumed to be shadowed (represented with a uniform distributed coefficient *U*, i.e., U∼U(0,1)) with a shadowing intensity $m_{s}$.

The resultant envelope probability density function $f_{R}\left(r\right)$, cumulative distribution function $F_{R}\left(r\right)$, and their instantaneous SNR counterparts (i.e., $f_{\Gamma }\left(\gamma \right),F_{\Gamma }\left(\gamma \right)$) are given by (see [18]):

(2) $$\left\{\begin{array}{c} f_{R}\left(r\right)=\frac{2m_{s}}{\Gamma (m)}r^{-1}G_{1,2}^{1,1}\left(\frac{m_{s}m}{(m_{s}-1)\Omega }r^{2}|\begin{array}{c} 1-m_{s}\\ m,-m_{s} \end{array}\right),\\ F_{R}\left(r\right)=\frac{m_{s}}{\Gamma (m)}G_{1,2}^{2,3}\left(\frac{m_{s}m}{(m_{s}-1)\Omega }r^{2}|\begin{array}{c} 1-m_{s},1\\ m,0,-m_{s} \end{array}\right), \end{array}\right.$$

(3) $$\left\{\begin{array}{c} f_{\Gamma }\left(\gamma \right)=\frac{2m_{s}}{\Gamma (m)}\gamma^{-1}G_{1,2}^{1,1}\left(\frac{m_{s}m}{\left(m_{s}-1\right)\bar{\gamma }}\gamma |\begin{array}{c} 1-m_{s}\\ m,-m_{s} \end{array}\right),\\ F_{\Gamma }\left(\gamma \right)=\frac{m_{s}}{\Gamma (m)}G_{1,2}^{2,3}\left(\frac{m_{s}m}{\left(m_{s}-1\right)\bar{\gamma }}\gamma |\begin{array}{c} 1-m_{s},1\\ m,0,-m_{s} \end{array}\right), \end{array}\right.$$

where $\Omega =\frac{2\sigma^{2}m_{s}m}{\left(m_{s}-1\right)}$ and the instantaneous SNR is related to the envelope as $\frac{\Gamma }{\bar{\gamma }}=\frac{R^{2}}{\Omega }$.

## 3. α-Fluctuating Nakagami Fading Channel Model

### 3.1. Physical Model

Considering the *a*-variate transformation procedure presented in [25], herein, it is assumed that the α-root envelope [25] follows the Fluctuating Nakagami distribution [18]:

(4) $$R^{\alpha }=\sum_{i=0}^{m}U^{-\frac{1}{m_{s}}}X_{i}^{2}+\sum_{i=0}^{m}U^{-\frac{1}{m_{s}}}Y_{i}^{2},$$

where *m* multi-path clusters (composed of independent zero-mean Gaussian components $X_{i},Y_{i}$ with equal variance σ) randomly fluctuate (i.e., U∼(0,1)) with shadowing severity $m_{s}>1$, and α, representing the nonlinear envelope distortion coefficient (due to nonhomogenity of the propagation environment [25]).

### 3.2. Statistical Model

Based on the given physical model (4), the statistical description of the α-Fluctuating Nakagami fading channel can be derived as follows.

 **Theorem 1.**  *For the α-Fluctuating Nakagami fading channel model, the first-order statistical characteristics (i.e., PDF $f_{\gamma }\left(\gamma \right)$, CDF $F_{\gamma }\left(\gamma \right)$, and MGF $M_{\gamma }\left(s\right)$) of the instantaneous SNR γ are given by:*

(5) $$\begin{array}{ccc} & & f_{\gamma }\left(\gamma \right)=\frac{\alpha m_{s}}{2\Gamma (m)}\gamma^{-1}G_{1,2}^{\;1,1}\;\left(\left.\frac{1}{℧}{\left(\frac{\gamma }{\bar{\gamma }}\right)}^{\frac{\alpha }{2}}\right|\;\begin{array}{c} 1-m_{s}\\ m,-m_{s} \end{array}\;\right), \end{array}$$

(6) $$\begin{array}{ccc} & & F_{\gamma }\left(\gamma \right)=\frac{m_{s}}{\Gamma (m)}H_{2,3}^{\;1,2}\;\left(\left.\frac{\gamma }{{℧}^{\frac{2}{\alpha }}\bar{\gamma }}\right|\;\begin{array}{c} \left(1,1\right),\left(1-m_{s},\frac{2}{\alpha }\right)\\ \left(m,\frac{2}{\alpha }\right),\left(-m_{s},\frac{2}{\alpha }\right),\left(0,1\right) \end{array}\;\right), \end{array}$$

(7) $$\begin{array}{ccc} & & \;M_{\gamma }\left(s\right)=\frac{m_{s}}{\Gamma (m)}H_{2,2}^{\;1,2}\;\left(\left.\frac{s^{-1}}{{℧}^{\frac{2}{\alpha }}\bar{\gamma }}\right|\;\begin{array}{c} \left(1,1\right),\left(1-m_{s},\frac{2}{\alpha }\right)\\ \left(m,\frac{2}{\alpha }\right),\left(-m_{s},\frac{2}{\alpha }\right) \end{array}\;\right), \end{array}$$

*where $℧={\left[\frac{\Gamma (m)}{m_{s}}\frac{\left(m_{s}-\frac{2}{\alpha }\right)}{\Gamma \left(m+\frac{2}{\alpha }\right)}\right]}^{\frac{\alpha }{2}}$; G(·),H(·) are the Meijer G- and the Fox H-function [46], where Γ(·) is the Euler gamma-function [46], and $\bar{\gamma }$ is the average SNR.*

**Proof** **of** **Theorem**  **1.**  To prove Theorem 1, one starts with the envelope PDF $f_{R}\left(r\right)$ of the underlying Fluctuating Nakagami model (see [18], Equation (7)). Confining oneself to the standard methodology of random variable transformation, first, one derives the PDF of $W=R^{2}$ (i.e., the power of the envelope) as $f_{W}\left(w\right)=\frac{1}{2}w^{-\frac{1}{2}}f_{R}\left(w^{\frac{1}{2}}\right)$. Secondly, perform the α-root transform of *W* (that is, $P^{\alpha }=W$) with PDF $f_{P}\left(\rho \right)=\alpha \rho^{\alpha -1}f_{W}\left(\rho^{\alpha }\right)$). Finally, denoting the second raw moment of the α-transformed power envelope as ψ (i.e., $\psi =E\left\{{\left(P\right)}^{2}\right\}$, and relating it to the instantaneous SNR γ as $f_{\gamma }\left(\gamma \right)=\frac{1}{2}\sqrt{\frac{\psi }{\gamma \bar{\gamma }}}f_{P}\left(\sqrt{\frac{\psi \gamma }{\bar{\gamma }}}\right)$, the required instantaneous SNR PDF of the α-Fluctuating Nakagami-*m* model can be related to the envelope PDF of the underlying Fluctuating Nakagami-*m* in the following form:

(8) $$f_{\gamma }\left(\gamma \right)=\frac{\alpha }{4}{\left(\frac{\psi }{\bar{\gamma }}\right)}^{\frac{\alpha }{4}}\gamma^{\frac{\alpha }{4}-1}f_{R}\left({\left[\frac{\psi }{\bar{\gamma }}\gamma \right]}^{\frac{\alpha }{4}}\right).$$

Combining the last result with ([18], Equation (7)) and evaluating $E\left\{{\left(P\right)}^{2}\right\}$ yields

(9) $$\psi =\frac{m_{s}}{\Gamma (m)}{\left(\frac{m_{s}m}{(m_{s}-1)\Omega }\right)}^{-\frac{2}{\alpha }}\frac{\Gamma \left(m+\frac{2}{\alpha }\right)}{\left(m_{s}-\frac{2}{\alpha }\right)},$$

which is valid if α>2; the PDF (5) is obtained.

The CDF (6), defined as $F_{\gamma }\left(\gamma \right)=\int_{0}^{\gamma }f_{\gamma }\left(x\right)dx$, can be obtained by relating the Meijer G-function and Fox H-function as

(10) $$\frac{\alpha }{2}G_{1,2}^{\;1,1}\;\left(\left.\frac{1}{℧}{\left(\frac{x}{\bar{\gamma }}\right)}^{\frac{\alpha }{2}}\right|\;\begin{array}{c} 1-m_{s}\\ m,-m_{s} \end{array}\;\right)=H_{2,3}^{\;1,2}\;\left(\left.\frac{x}{{℧}^{\frac{2}{\alpha }}\bar{\gamma }}\right|\;\begin{array}{c} \left(1-m_{s},\frac{2}{\alpha }\right)\\ \left(m,\frac{2}{\alpha }\right),\left(-m_{s},\frac{2}{\alpha }\right) \end{array}\;\right)$$

and utilizing ([47], Equation (2.8.18)).

The MGF (7), defined as

(11) $$M_{\gamma }\left(s\right)=E\left\{e^{s\gamma }\right\}$$

can be evaluated with the help of the derived CDF, i.e.,

(12) $$M_{\gamma }\left(-s\right)=s\int_{0}^{\infty }F_{\gamma }\left(\gamma \right)e^{-s\gamma }d\gamma .$$

Treating the integral as the Laplace transform L{f(γ)}(s) of a function f(γ) evaluated at *s*, applying the identity (see [47], Theorem 2.3, Equation (2.5.16)):

(13) $$\left.L\left\{H_{p,q}^{m,n}\left[\gamma \left|\begin{array}{c} {\left(a_{i},\alpha_{i}\right)}_{1,p}\\ {\left(b_{j},\beta_{j}\right)}_{1,q} \end{array}\right.\right.\right]\right\}\left(s\right)=\frac{1}{t}H_{p+1,q}^{m,n+1}\left[\frac{1}{s}\left|\begin{array}{c} \left(0,1\right),{\left(a_{i},\alpha_{i}\right)}_{1,p}\\ {\left(b_{j},\beta_{j}\right)}_{1,q} \end{array}\right.\right],$$

and simplifying the expression via the reduction property ([47], Equation (2.1.1)) finalizes the proof of (7). □

It must be highlighted that (5)–(7) are valid ∀m≥0.5 and $m_{s},\alpha$ that satisfy the condition $\frac{m_{s}\alpha }{2}>1$ (as to the expression for ℧). It is immediately observed that substitution α=2 in (5) and (6) after some simplifications results in equivalent expressions for the underlying Fluctuating Nakagami model (see [18], Equations (12) and (13)).

It is important to observe that the special functions referenced in Theorem 1 and all that follows, as well as the accompanying Corollaries, are easily accessible within modern computer algebra systems (CAS) (e.g., Wolfram Mathematica, MATLAB, Maple, etc.) for both symbolic and numerical computations. Additionally, further numerical simulations indicate that, in almost all scenarios of practical interest, these functions can be computed with high efficiency through univariate numerical integration in the complex domain speeding up the computations, in addition to utilizing the existing CAS methods. For example, in the case of the MGF (7), it can be rewritten in the following form:

(14) $$M_{\gamma }\left(s\right)=\frac{m_{s}}{\Gamma (m)}\frac{1}{2\pi i}\int_{L}\frac{\Gamma \left(-\sigma \right)\Gamma \left(m+\frac{2\sigma }{\alpha }\right)}{\left(m_{s}-\frac{2\sigma }{\alpha }\right)}{\left(\frac{s^{-1}}{{℧}^{\frac{2}{\alpha }}\bar{\gamma }}\right)}^{-\sigma }d\sigma ,$$

where L is the contour chosen in such a way as to separate the poles Γ(−σ) and $\Gamma \left(m+\frac{2\sigma }{\alpha }\right)$. For example, it can be chosen as $L=\left(-\frac{\alpha m}{4}-i\infty ;-\frac{\alpha m}{4}+i\infty \right)$.

From the practical standpoint, it is important to have at hand the channel statistics for infinitely increasing SNR (the so-called high-SNR asymptotics) since it is needed to upper-bound the channel performance. The derived expressions in the high-SNR region can be summarized as follows.

 **Corollary** **1.**  *The high-SNR asymptotics (i.e., $\bar{\gamma }\to \infty$) of PDF, CDF, and MGF for the α-Fluctuating Nakagami model are given by*

(15) $$\begin{array}{ccc} f_{\gamma }\left(\gamma \right){|}_{\bar{\gamma }\to \infty } & ⋍ & \frac{m_{s}{℧}^{-m}}{\left(m_{s}+m\right)\Gamma \left(m\right)\gamma }{\left(\frac{\gamma }{\bar{\gamma }}\right)}^{\frac{\alpha m}{2}}, \end{array}$$

(16) $$\begin{array}{ccc} F_{\gamma }\left(\gamma \right){|}_{\bar{\gamma }\to \infty } & ⋍ & \frac{m_{s}{℧}^{-m}}{\left(m_{s}+m\right)\Gamma \left(m+1\right)}{\left(\frac{\gamma }{\bar{\gamma }}\right)}^{\frac{\alpha m}{2}}, \end{array}$$

(17) $$\begin{array}{ccc} M_{\gamma }\left(s\right){|}_{\bar{\gamma }\to \infty } & ⋍ & \frac{\alpha m_{s}\Gamma \left(\frac{\alpha m}{2}\right)}{2\left(m_{s}+m\right)\Gamma \left(m\right)}\frac{{℧}^{-m}}{{\left(s\bar{\gamma }\right)}^{\frac{\alpha m}{2}}}. \end{array}$$

**Proof.**  The expression (15)–(17) can be proved by observing that for a vanishingly small argument (i.e., for $\bar{\gamma }\to \infty$), the Fox H-functions can be approximated by the single terms of their power-series expansions, i.e. (see [47], Corollary 1.11.1),

(18) $$H_{p,q}^{\;m,n}\;\left(\left.\frac{x}{\bar{\gamma }}\right|\;\begin{array}{c} {\left(a_{i},A_{i}\right)}_{i=1,p}\\ {\left(b_{i},B_{i}\right)}_{j=1,q} \end{array}\;\right)∼h_{j_{0}}{\left(\frac{x}{\bar{\gamma }}\right)}^{\frac{b_{j_{0}}}{B_{j_{0}}}},$$

where for all the assumed cases (i.e., PDF, CDF, and MGF (5)–(7)) $j_{0}=1$, $\frac{b_{j_{0}}}{B_{j_{0}}}=\frac{\alpha m}{2}$, and $h_{j_{0}}$ is evaluated in each case according to ([47], Equation (1.8.5)). Simplifying the obtained expression yields (15)–(17). □

To complete the first-order statistical description, required for further channel performance assessment, the instantaneous SNR raw moments must be derived.

 **Theorem 2.**  *The $k^{\underline{th}}$ raw moment of the instantaneous SNR for the α-Fluctuating Nakagami fading channel model can be expressed as:*

(19) $$\begin{array}{ccc} E\{\gamma^{k}\} & = & \frac{m_{s}}{\Gamma (m)}{℧}^{\frac{2k}{\alpha }}\frac{\Gamma \left(m+\frac{2k}{\alpha }\right)}{\left(m_{s}-\frac{2k}{\alpha }\right)}{\bar{\gamma }}^{k}. \end{array}$$

**Proof.**  The proof is obtained by combining the definition of the SNR $k^{\underline{th}}$ raw moment, i.e., $E\left\{\gamma^{k}\right\}=\int_{0}^{\infty }\gamma^{k}f_{\gamma }\left(\gamma \right)d\gamma$, with the derived PDF (5) in the following way:

(20a) $$\begin{array}{cccc} E\{\gamma^{k}\} & & = & \;\int_{0}^{\infty }\gamma^{k}f_{\gamma }\left(\gamma \right)d\gamma \end{array}$$

(20b) $$\begin{array}{cccc} & & = & \;\frac{\alpha m_{s}}{2\Gamma (m)}\int_{0}^{\infty }\gamma^{k-1}G_{1,2}^{\;1,1}\;\left(\left.\frac{1}{℧}{\left(\frac{\gamma }{\bar{\gamma }}\right)}^{\frac{\alpha }{2}}\right|\;\begin{array}{c} 1-m_{s}\\ m,-m_{s} \end{array}\;\right)d\gamma = \end{array}$$

(20c) $$\begin{array}{cccc} & & = & \;\frac{m_{s}}{2\Gamma (m)}\int_{0}^{\infty }\gamma^{k-1}H_{1,2}^{\;1,1}\;\left(\left.\frac{\gamma }{{℧}^{\frac{2}{\alpha }}\bar{\gamma }}\right|\;\begin{array}{c} \left(1-m_{s},\frac{2}{\alpha }\right)\\ \left(m,\frac{2}{\alpha }\right),\left(-m_{s},\frac{2}{\alpha }\right) \end{array}\;\right)d\gamma = \end{array}$$

(20d) $$\begin{array}{cccc} & & = & \;\frac{m_{s}}{\Gamma (m)}{℧}^{\frac{2k}{\alpha }}\frac{\Gamma \left(m+\frac{2k}{\alpha }\right)}{\left(m_{s}-\frac{2k}{\alpha }\right)}{\bar{\gamma }}^{k}, \end{array}$$

where the transition from (20b) to (20d) was performed via ([47], Equation (2.5.7)).

It must be pointed out that the expression (19) is valid for $\frac{m_{s}\alpha }{2k}>1$. □

The derived expression of the SNR moments helps to evaluate such channel characteristic as amount of fading, which is generally used to describe the fading severity and is defined as the normalized variance of the SNR, i.e., $\mathrm{AoF}=\frac{E\{\gamma^{2}\}}{{\left(E\{\gamma \}\right)}^{2}}-1$.

 **Corollary** **2.**  *The amount of fading for the α-Fluctuating Nakagami model is given by*

(21) $$\mathrm{AoF}=\frac{{\left(m_{s}-\frac{2}{\alpha }\right)}^{2}}{m_{s}\left(m_{s}-\frac{4}{\alpha }\right)}\frac{\Gamma \left(m\right)\Gamma \left(m+\frac{4}{\alpha }\right)}{{\left(\Gamma \left(m+\frac{2}{\alpha }\right)\right)}^{2}}-1$$

The proof of Corollary 2 is omitted since it trivially follows from the definition of AoF and the derived expression for the raw moments (19) with k=2.

The provided first-order statistical description of the α-Fluctuating Nakagami fading channel model facilitates the evaluation of general metrics describing the overall system performance.

## 4. Performance Analysis of the α-Fluctuating Nakagami Model

It is commonly accepted to assess the performance of a WSN communication that operates in the presence of a fading channel in terms of the outage probability (OP), average bit error rate (ABER), and ergodic capacity (EC), which are defined as follows. For the α-Fluctuating Nakagami model, these metrics derived in closed form as follows.

### 4.1. Outage Analysis of the α-Fluctuating Nakagami Model

The outage probability is defined as the probability of the event that the instantaneous SNR falls below the specified threshold ($\gamma_{th}$), i.e., $P_{out}=P\left\{\gamma <\gamma_{th}\right\}$. This is exactly the cumulative distribution derived in Theorem 1, i.e., $P_{out}=F_{\gamma }\left(\gamma_{th}\right)$, with the asymptotic performance presented in Corollary 1.

### 4.2. ABER Performance of the α-Fluctuating Nakagami Model

For a broad variety of coherent modulations widely used in wireless communications [48] (e.g., BPSK, GMSK (for high $\bar{\gamma }$), M-PSK, square M-QAM, etc.), ABER can be efficiently approximated as a combination of Gauss Q-functions Q(·), i.e.,

(22) $${\bar{P}}_{er}^{c}=\delta_{1}\sum_{j=1}^{\delta_{3}}\int_{0}^{\infty }Q\left(\sqrt{2\delta_{2,j}\gamma }\right)f_{\gamma }\left(\gamma \right)d\gamma ,$$

where the combination of coefficients $\left\{\delta_{1},\delta_{2,j},\delta_{3}\right\}$ are properly defined for the particular modulation. For example, in the case of M-QAM: 41−1Mlog2M,3(2j−1)22(M−1),M2 and for M-PSK: $\left\{\frac{1}{\max(2,\log_{2}M)},2\log_{2}M\sin^{2}\left(\frac{(2j-1)\pi }{M}\right),\max\left(1,\frac{M}{4}\right)\right\}$.

For several non-coherent modulations, ABER can be efficiently approximated by [48]:

(23) $${\bar{P}}_{er}^{nc}=\sum_{j=1}^{\varepsilon_{3}}\varepsilon_{1,j}\int_{0}^{\infty }e^{-\varepsilon_{2,j}\gamma }f_{\gamma }\left(\gamma \right)d\gamma ,$$

where, for example, $\left\{\varepsilon_{1,j},\varepsilon_{2,j},\varepsilon_{3}\right\}$ are equal to (−1)j+1j+1M−1j,jj+1,M−1 for M-FSK and $\left\{\frac{1}{2},1,1\right\}$ for DBPSK.

Applying the results of the derived statistical representation, the ABER can be expressed in closed form as follows.

 **Theorem** **3.**  *For the α-Fluctuating Nakagami fading channel model, the average bit error rate can be expressed as:*

- *For the coherent modulations (e.g., M-QAM and M-PSK):*
(24) $${\bar{P}}_{er}^{c}=\frac{\delta_{1}m_{s}}{2\sqrt{\pi }\Gamma \left(m\right)}\sum_{j=1}^{\delta_{3}}H_{3,3}^{\;1,3}\left(\left.\frac{\delta_{2,j}^{-1}}{{℧}^{\frac{2}{\alpha }}\bar{\gamma }}\right|\;\begin{array}{c} \left(\;\frac{1}{2},1\right),\left(1,1\right),\left(1-m_{s},\frac{2}{\alpha }\right)\\ \left(m,\frac{2}{\alpha }\right),\left(-m_{s},\frac{2}{\alpha }\right),\left(0,1\right) \end{array}\;\right),$$
- *For the non-coherent modulations (e.g., DBPSK, BFSK, and M-FSK):*
(25) $${\bar{P}}_{er}^{nc}=\frac{m_{s}}{\Gamma (m)}\sum_{j=1}^{\varepsilon_{3}}\varepsilon_{1,j}H_{3,3}^{\;1,3}\left(\left.\frac{{℧}^{-\frac{2}{\alpha }}}{\varepsilon_{2,j}\bar{\gamma }}\right|\;\begin{array}{c} \left(0,1\right),\left(1,1\right),\left(1-m_{s},\frac{2}{\alpha }\right)\\ \left(m,\frac{2}{\alpha }\right),\left(-m_{s},\frac{2}{\alpha }\right),\left(0,1\right) \end{array}\;\right),$$

**Proof.**  To prove Theorem 3, one can note that, via integration-by-parts, (22) is expressed as:

(26) $$\int_{0}^{\infty }Q\left(\sqrt{2\delta_{2,j}\gamma }\right)f_{\gamma }\left(\gamma \right)d\gamma =\sqrt{\frac{\delta_{2,j}}{4\pi }}\int_{0}^{\infty }\frac{e^{-\delta_{2,j}\gamma }}{\sqrt{\gamma }}F_{\gamma }\left(\gamma \right)d\gamma .$$

The latter integral can be regarded as the Laplace transform L{·,s} evaluated at $s=\delta_{2,j}$:

(27) $${\bar{P}}_{er}^{c}=\frac{\delta_{1}}{2\sqrt{\pi }}\sum_{j=1}^{\delta_{3}}\delta_{2,j}^{-\frac{1}{2}}L\left\{\frac{F_{\gamma }\left(\gamma \right)}{\sqrt{\gamma }};\delta_{2,j}\right\}.$$

Applying ([47], Corollary 2.3.1, Equation (2.5.25)), after some simplifications, (26) follows. For non-coherent modulations, using the same strategy as in (26) yields:

(28) $${\bar{P}}_{er}^{nc}=\sum_{j=1}^{\varepsilon_{3}}\varepsilon_{1,j}\varepsilon_{2,j}L\left\{F_{\gamma }\left(\gamma \right);\varepsilon_{2,j}\right\}.$$

Noting that the Laplace transform of the CDF can be related to the MGF (i.e., $L\left\{F_{\gamma }\left(\gamma \right);\varepsilon_{2,j}\right\}=\varepsilon_{2,j}^{-1}M_{\gamma }\left(\varepsilon_{2,j}\right)$), after applying (7) and some straightforward modifications, (27) is obtained, finalizing the proof. □

The resultant ABER expressions are derived in closed form and valid for arbitrary channel parameters, but from the practical standpoint, it is important to understand ABER high-SNR behavior, since it bounds from above the performance quality.

Applying the results of Corollary 1, the high-SNR behavior of (15)–(17) can be obtained as follows.

 **Corollary** **3.**  *The high-SNR asymptotics of the average bit error rate expressions of the ABER (24) and (25) are given by:*

- *for the coherent modulations:*
(29) $${\bar{P}}_{er}^{c}{|}_{\bar{\gamma }\to \infty }\approx \frac{\delta_{1}m_{s}\Gamma \left(\frac{\alpha m+1}{2}\right)}{2\sqrt{\pi }\Gamma \left(m+1\right)\left(m_{s}+m\right){℧}^{m}}{\bar{\gamma }}^{-\frac{\alpha m}{2}}\sum_{j=1}^{\delta_{3}}\delta_{2,j}^{-\frac{\alpha m}{2}},$$
- *for the non-coherent modulations:*
(30) $${\bar{P}}_{er}^{nc}{|}_{\bar{\gamma }\to \infty }\approx \frac{\alpha m_{s}\Gamma \left(\frac{\alpha m}{2}\right)}{2\left(m_{s}+m\right)\Gamma \left(m\right){℧}^{m}}{\bar{\gamma }}^{-\frac{\alpha m}{2}}\sum_{j=1}^{\varepsilon_{3}}\varepsilon_{1,j}{\left(\varepsilon_{2,j}\right)}^{-\frac{\alpha m}{2}}.$$

In wireless communications through fading channels, it is a common practice to describe the possible performance improvements due to channel conditions in terms of the so-called diversity gain $G_{D}$ and the coding gain $G_{C}$, which are defined via the asymptotic ABER expression in the following form ${\bar{P}}_{er}{|}_{\bar{\gamma }\to \infty }={\left(G_{C}\bar{\gamma }\right)}^{-G_{D}}$.

 **Corollary** **4.**  *The diversity gain for the α-Fluctuating Nakagami model linearly scales with the envelope transformation coefficient α, and does not depend on the shadowing coefficient $m_{s}$, i.e., $G_{d}=\frac{\alpha m}{2}$ for both coherent and non-coherent modulations.*

### 4.3. Capacity Analysis of the α-Fluctuating Nakagami Model

The channel capacity of the wireless communication link in the presence of multipath fading effects is commonly defined as

(31) $$\bar{C}=\int_{0}^{\infty }\log_{2}\left(1+\gamma \right)f_{\gamma }\left(\gamma \right)d\gamma .$$

The obtained statistical description of the α-Fluctuating Nakagami channel model presented in Section 2 helps to derive the exact and asymptotic expressions for $\bar{C}$.

 **Theorem 4.**  *The average channel capacity of the α-Fluctuating Nakagami model can be expressed as*

(32) $$\bar{C}=\frac{m_{s}}{\Gamma (m)\ln2}H_{3,4}^{\;3,2}\left(\left.\frac{{℧}^{-\frac{2}{\alpha }}}{\bar{\gamma }}\right|\;\begin{array}{c} \left(0,1\right),\left(1-m_{s},\frac{2}{\alpha }\right),\left(1,1\right)\\ \left(m,\frac{2}{\alpha }\right),\left(0,1\right),\left(0,1\right),\left(-m_{s},\frac{2}{\alpha }\right) \end{array}\;\right).$$

**Proof.**  To prove Theorem 4, one starts with applying the contour integral representation of $G_{1,2}^{\;1,1}\left(\cdot \right)$ in (5) and rearranging the integration order:

(33) $$\bar{C}=\frac{m_{s}\log_{2}e}{\Gamma (m)}\frac{1}{2\pi i}\int_{H}\frac{\Gamma \left(m+\frac{2s}{\alpha }\right)\Gamma \left(m_{s}-\frac{2s}{\alpha }\right)}{\Gamma \left(m_{s}+1-\frac{2s}{\alpha }\right)}\left(\int_{0}^{\infty }\frac{\ln(1+\gamma )}{\gamma^{s+1}}d\gamma \right){\left(\frac{1}{{℧}^{\frac{2}{\alpha }}\bar{\gamma }}\right)}^{-s}ds.$$

The inner integral over γ via ([49], Equation (2.6.9.21)) can be expressed as $\frac{\pi \csc(\pi s)}{s}$ (for 0<ℜ{s}<1). Using the reflection and recurrence property of the gamma-function (see [46], Equation (5.5.3) and [46], Equation (5.5.1), respectively), which relate it to the cosecant function, helps to represent the inner integral solely in terms of the gamma-functions, i.e., $\frac{\pi \csc(\pi s)}{s}=\frac{\Gamma^{2}\left(s\right)\Gamma \left(1-s\right)}{\Gamma (1+s)}$. Lastly, one can shift the integration contour H in such a way that $0<ℜ\left\{s\right\}<\frac{m_{s}\alpha }{2}$ and note that the obtained integral and its integration contour is a valid Mellin–Barnes representation of the Fox H-function in (32), which finalizes the proof. □

 **Corollary** **5.**  *The high-SNR approximation of the average capacity for the α-Fluctuating Nakagami model is given by*

(34) $$\bar{C}{|}_{\bar{\gamma }\to \infty }\approx \log_{2}\left({℧}^{\frac{2}{\alpha }}\bar{\gamma }\right)+\frac{2}{\alpha m_{s}\ln2}\left(1+m_{s}\psi^{\left(0\right)}\left(m\right)\right),$$

*where $\psi^{\left(0\right)}\left(\cdot \right)$ is the digamma-function [46].*

**Proof.**  The asymptotic channel capacity is obtained by noting that (see [17], Equations (6) and (7)):

(35) $$\bar{C}{|}_{\bar{\gamma }\to \infty }\approx \log_{2}\gamma +\frac{1}{\ln2}\frac{d}{dk}\left(\frac{E\{\gamma^{k}\}}{{\left(E\left\{\gamma \right\}\right)}^{k}}\right){|}_{k=0}.$$

Applying Theorem 2, performing differentiation and limiting operation with k→0, after some simplifications, (34) follows. □

To the best of the author’s knowledge, the α-Fluctuating Nakagami fading model has not been reported in the technical literature yet, and the derived results of its statistical description are novel.

## 5. Simulation and Results’ Analysis

To prove the correctness of the performed analytical work, numerical simulations as well as experimental verification were performed. To this extent, the results obtained in Section 3 and Section 4 (see Theorems 1–4 and the respective corollaries) were used to evaluate the corresponding characteristics (i.e., PDF, CDF, MGF, raw moments, AoF, outage probability, ABER, and EC) analytically, and were compared with the ones obtained via brute-force numerical integration (used in their definitions). All results were accompanied (where applicable) by the corresponding derived high-SNR asymptotics (depicted with dashed blue lines) and Monte Carlo simulation with $10^{5}$ samples.

Since channel performance is completely defined by the set of channel parameters, they were chosen in such a way as to correspond to as many practical scenarios as possible, covering both heavy and light fading (described by *m*), shadowing (controlled by $m_{s}$), and nonlinear distortions (α).

One has to emphasize the limitations implied on the parameters. It is clear that, due to the initial physical model (see Section 3.1), no limitations are implied on the nonlinearity coefficient α (except for positivity, i.e., α>0), but the shadowing coefficient is assumed to be $m_{s}>1$ (due to the limitations of the original model). Moreover, the results derived in Section 3 and Section 4 are evaluated with restriction $\frac{m_{s}\alpha }{2}>1$ in the cases where first-order moments are used, and $\frac{m_{s}\alpha }{2k}>1$ in the cases where *k*-order moments are needed. The restriction on the fading parameter *m* is generally due to the fact that the baseline model is induced by the basic Nakagami-*m* channel, thus m≥0.5.

### 5.1. Model Experimental Verification and Positioning

**Figure 1:**
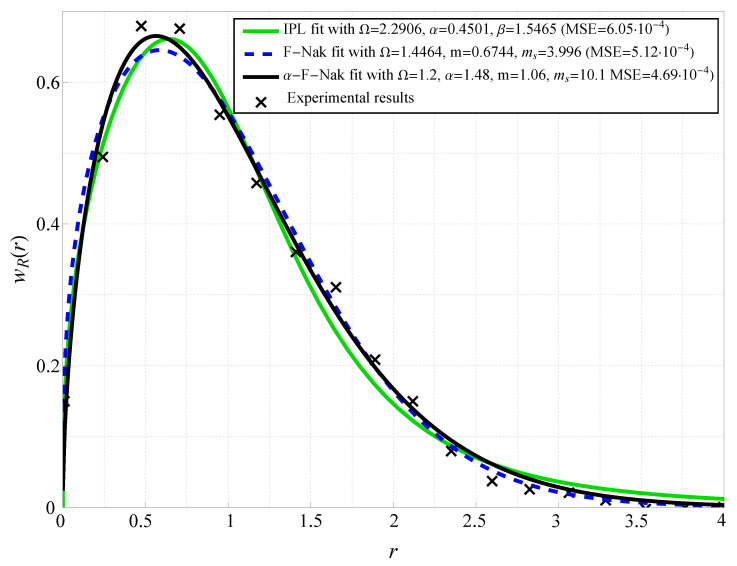
PDF fitting with experimental data from D2D measurements (see [50]). Fluctuating Nakagami (F-Nak) is fitted with the parameters presented in [18], and Inverse Power Lomax (IPL) with parameters presented in [17].

Figure 1 presents the probability density function (PDF) fitting of the α-Fluctuating Nakagami-*m* model against real-world experimental data obtained from device-to-device (D2D) wireless measurements. The proposed model is compared with two conventional fading models: the Fluctuating Nakagami-*m* (denoted as F-Nak) model and the Inverse Power Lomax (denoted as IPL) model. The plot shows that the α-Fluctuating Nakagami-*m* model achieves the closest match to empirical data across a wide range of signal variations. The fitting quality is quantitatively assessed using the mean squared error (MSE), where the proposed model exhibits the lowest MSE value of approximately $4.69\times 10^{-4}$, compared to $5.12\times 10^{-4}$ for F-Nak and $6.05\times 10^{-4}$ for IPL. This indicates that the α-Fluctuating Nakagami-*m* model provides a more flexible statistical representation of real-world fading conditions. The figure further illustrates that traditional models tend to underestimate the probability of deep fades, particularly in the hyper-Rayleigh region, whereas the proposed model accurately captures these effects. The fitting curves show that the deviation between the theoretical and empirical distributions is minimal at moderate signal levels, confirming the robustness of the statistical framework. The high accuracy of the proposed model confirms its applicability in modern wireless communication system analysis, particularly in environments characterized by severe multipath effects.

**Figure 2:**
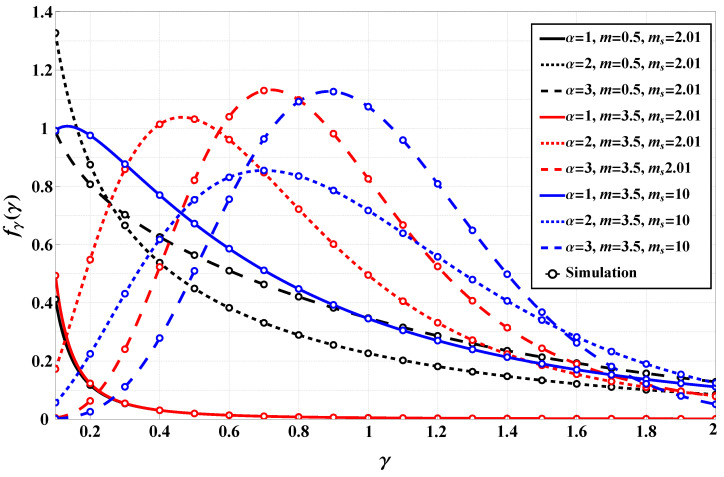
PDF comparison of numerical evaluation and numerical simulation across various channel parameters.

Figure 2 presents a detailed comparison between analytically derived and simulated probability density functions (PDFs) of the α-Fluctuating Nakagami-*m* model under various channel parameters. The figure contrasts numerically evaluated PDFs (using the derived expressions) with results obtained from Monte Carlo simulations, validating the theoretical framework. Multiple parameter sets are illustrated to cover different fading conditions, as was stated earlier. For instance, for m=3.5, α=1, and $m_{s}=10$, the simulated and numerical curves align almost perfectly, demonstrating a robust model behavior under low shadowing conditions. In contrast, for m=0.5 and $m_{s}=2$, a scenario associated with heavier fading, the PDFs exhibit longer tails, yet maintain high consistency between theory and simulation. This consistency across a wide range of parameters affirms the model’s stability and accuracy. Compared to Figure 1, which focused on empirical fitting with real-world D2D data, Figure 2 emphasizes the model’s analytical integrity through synthetic validation. While Figure 1 highlighted the α-FN model’s superiority in empirical fitting over other fading models like IPL and Fluctuating Nakagami-*m*, Figure 2 further confirms that the theoretical framework is computationally sound and matches stochastic behavior even in challenging fading environments. These results support the model’s usability for performance prediction across practical wireless scenarios.

**Figure 3:**
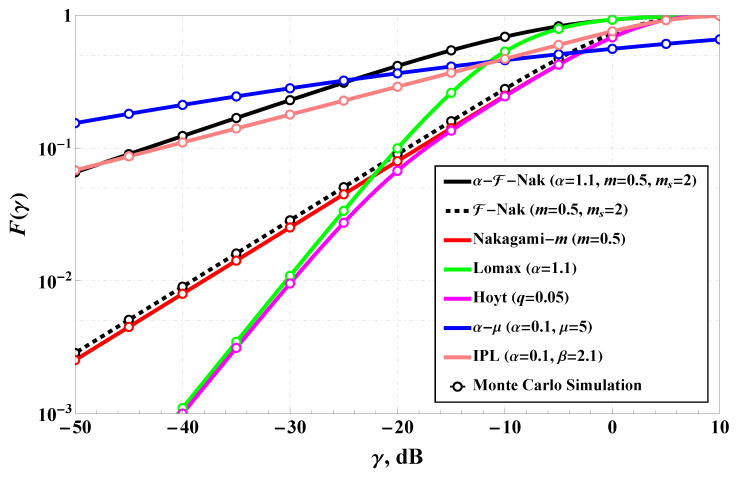
α-Fluctuating Nakagami-*m* model positioning (in terms of CDF) among different multipath channels: Nakagami-*m* [48], Fluctuating Nakagami-*m* [18], Inverse Power Lomax [17], Hoyt [48], Lomax [16], and α−μ [25].

Figure 3 illustrates the positioning of the α-Fluctuating Nakagami-*m* (α-FN) model in terms of its cumulative distribution function (CDF), compared to several well-known multipath fading models. These include the standard Nakagami-*m* model [48], Fluctuating Nakagami-*m* [18], Inverse Power Lomax [17], Hoyt [48], Lomax [16], and α−μ [25] fading models. The parameters are fixed such that α=1.1, m=0.5, and $m_{s}=2$ for the α-FN and Fluctuating Nakagami-*m* models, with appropriate configurations for the others to ensure a fair comparison. The figure clearly shows that the α-FN model provides a unique behavior that interpolates between light-tailed and heavy-tailed fading distributions. In particular, the CDF of the α-FN model closely resembles that of hyper-Rayleigh fading, but with better adaptability across various regimes. The plot demonstrates that at low SNRs, the α-FN model’s curve rises more gradually than Nakagami-*m* and Fluctuating Nakagami-*m*, indicating a heavier tail and higher probability of deep fades. Compared to the IPL and Hoyt models, the α-FN distribution remains flexible, providing a better fit for a wide range of fading environments. This adaptability reinforces its value as a unified fading model capable of encompassing diverse real-world propagation characteristics. Monte Carlo simulations align well with the analytical CDF, validating the accuracy of the derived expressions. Overall, Figure 3 supports the conclusion that the α-FN model generalizes and enhances traditional models by offering increased tunability and closer alignment with empirical fading statistics.

**Figure 4:**
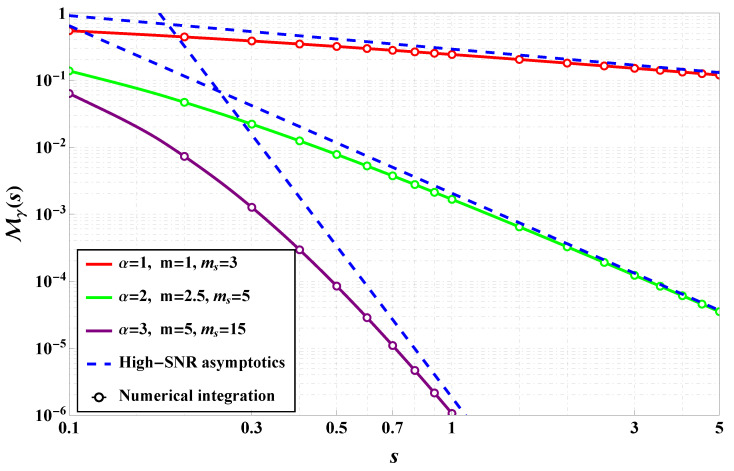
MGF comparison of numerical evaluation and numerical simulation across various channel parameters.

As was mentioned, MGF is one of the key analytical tools used to further evaluate performance metrics such as average bit error rate and outage probability. In Figure 4, the solid lines represent the exact analytical expressions for the MGF derived using Fox’s H-function-based formulations, while the markers correspond to numerical integration results that validate the theoretical expressions. The plot illustrates how the MGF behaves under varying values of the α parameter, which controls the degree of nonlinearity in the fading envelope, as well as the *m* and $m_{s}$ parameters that govern fading severity and shadowing intensity, respectively. It is evident that the rate of decay of the MGF curve varies significantly depending on the chosen parameters. For lower values of α, such as α = 0.1, the MGF decays more slowly, suggesting higher variability in the channel and a greater likelihood of deep fades. This aligns with the interpretation that smaller α values correspond to more severe nonlinear distortions in the propagation environment. Increasing the fading severity parameter *m* leads to a faster decay of the MGF, consistent with the behavior of the classical Nakagami-m model where larger *m* values correspond to less severe fading conditions. Similarly, increasing the shadowing parameter $m_{s}$ results in slower MGF decay, indicating that stronger shadowing causes fading effects even at high SNR levels. The close match between the analytical curves and the numerical integration results confirms the correctness of the derived MGF expressions and supports their use in subsequent performance analysis. This also demonstrates the flexibility of the α-Fluctuating Nakagami-m model in capturing both light and heavy fading scenarios by adjusting the α parameter, thereby extending the applicability beyond conventional models. The results indicate that the MGF can be effectively used for deriving the system-level performance bounds (studied further) for wireless communication systems operating in complex propagation environments such as device-to-device (D2D) links, millimeter-wave (mmWave), and terahertz (THz) channels. Furthermore, the parametric control offered by α allows for better fitting to empirical data in real-world scenarios where traditional models may fail to capture observed fading dynamics. The numerical validation reinforces the robustness of the analytical framework and supports its use in practical applications such as link budget calculations, adaptive modulation design, and diversity combining techniques.

### 5.2. α-Fluctuating Nakagami-*m* Model Performance

The current section presents the results of the numerical analysis of system-level performance metrics (i.e., outage probability, average error rate, and ergodic capacity) derived in closed-form in Section 4.

**Figure 5:**
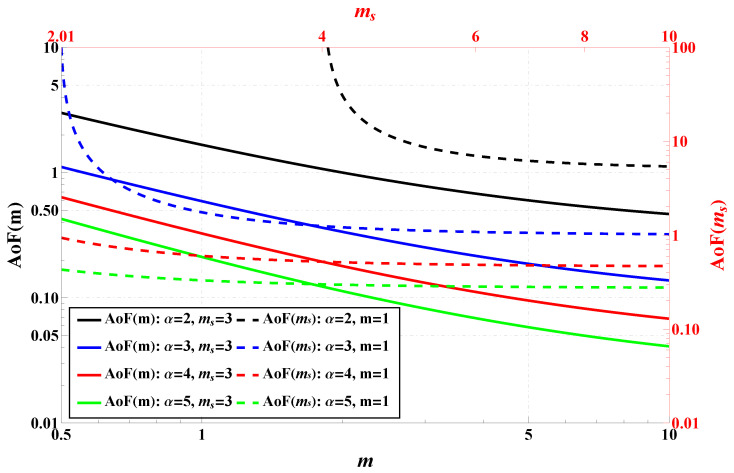
AoF comparison for various channel parameters.

One starts the system performance assessment in terms of the Amount of Fading plotted in Figure 5 across various channel parameters. As was mentioned, AoF serves as a key metric to quantify the severity of fading in wireless channels. The figure combines two plots. The right-hand vertical axis encompasses $\mathrm{AoF}(m_{s})$ (the results are plotted with dashed lines) and the left-hand AoF(m) (the results are plotted with solid lines). Qualitatively, the left plot shows that as *m* increases, indicating reduced multipath fading, the AoF systematically decreases for all values of α. This trend confirms that higher *m* contributes to more stable wireless channels. Quantitatively, when α=3, the AoF decreases from approximately 1 at m=0.5 to less than 0.2 at m=10, highlighting an almost order of magnitude reduction in fading severity. A similar pattern is observed for other values of α. In particular, the decline in AoF (as *m* increases) is roughly the same for different values of α, indicating that although the nonlinearity of the model helps suppress the effects of fading (in absolute terms), the rate of improvements (in relative terms) depends weakly on α.

The right-hand plot analyzes AoF as a function of $m_{s}$, where increasing $m_{s}$ corresponds to weaker shadowing. It shows that AoF significantly drops as $m_{s}$ increases from roughly 2 to 10, especially for smaller α. For example, when α=3, AoF reduces from about 100 at $m_{s}=2$ to roughly 1 at $m_{s}=10$, demonstrating a substantial improvement in channel stability. Cross-comparison between the plots reveals that both *m* and $m_{s}$ contribute to reducing AoF, but their impact is determined by the value of α. The effect of α is more pronounced at lower *m* and $m_{s}$, making it particularly useful in harsh fading conditions.

**Figure 6:**
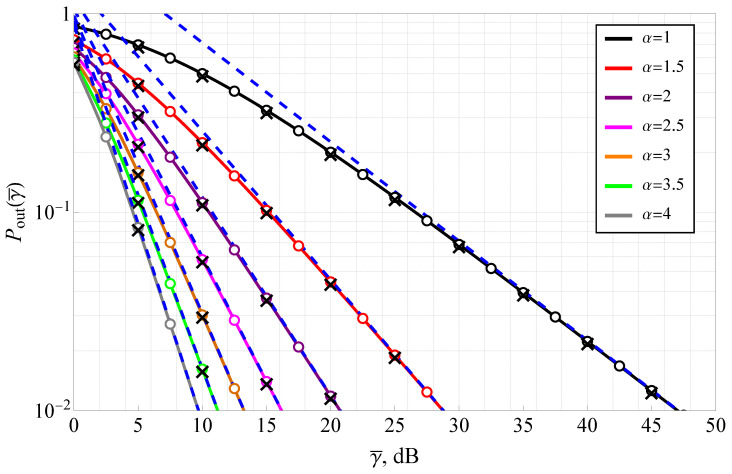
Outage probability for various α with m=1, $m_{s}=2.5$, and $\gamma_{th}=0$ dB: solid lines—the derived analytic solutions (6), dashed blue lines—the derived asymptotic expression (16), circle markers—numeric integration, black cross markers—Monte Carlo simulation.

Figure 6 illustrates the outage probability as a function of signal-to-noise ratio (SNR) for various values of the transformation parameter α, with fixed parameters m=1, $m_{s}=2.5$, and a threshold SNR of $\gamma_{th}=0$ dB. The outage probability is a critical metric that quantifies the likelihood of the instantaneous SNR falling below a predefined threshold, directly impacting link reliability in wireless communication systems. The plot demonstrates that increasing α leads to a significant reduction in outage probability, highlighting the role of α in mitigating extreme fading effects. At $\bar{\gamma }=25$ dB, the outage probability for α=1 is approximately $10^{-1}$, whereas for α=4, it decreases to nearly $10^{-4}$. This implies that higher α values lead to an order-of-magnitude improvement in link reliability at moderate SNRs. The numerical integration results (circle markers) and Monte Carlo simulations (cross markers) closely match the analytically derived outage probability (solid lines), validating the theoretical formulation. The high-SNR asymptotic expressions (dashed lines) provide excellent approximations for small α (i.e., α<2) beyond $\bar{\gamma }=20$ dB, and for α>2, beyond 10 dB, simplifying performance evaluation at practical operational SNR levels. Notably, for low α, the outage probability saturates at high SNRs, indicating that deep fading events remain dominant despite increasing transmission power. This effect is particularly pronounced in the hyper-Rayleigh regime, reinforcing the necessity of advanced signal processing techniques for mitigating fading-induced performance degradation.

**Figure 7:**
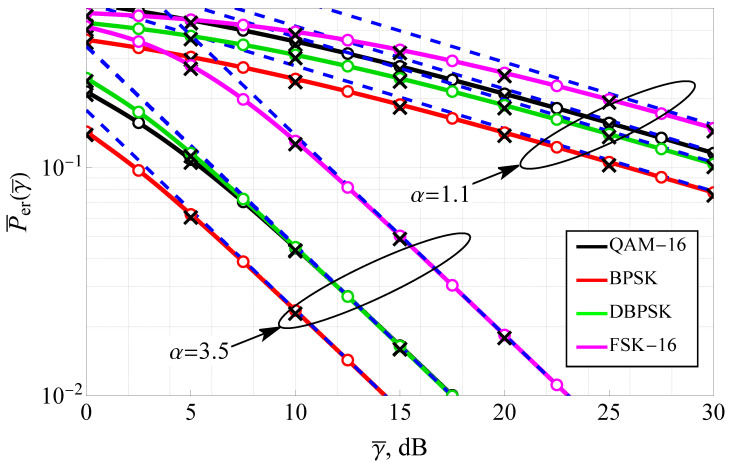
ABER for coherent and non-coherent modulations for various α with $m=\frac{1}{2}$, $m_{s}=2$: solid lines—the derived analytic solutions (24) and (25), dashed blue lines—the derived asymptotic expression (29) and (30), circle markers—numeric integration (22) and (23), black cross markers—Monte Carlo simulation.

Figure 7 presents the average bit error rate (ABER) as a function of SNR for both coherent and non-coherent modulations, evaluated under different α values with m=0.5 and $m_{s}=2$. The figure includes results for widely used modulation schemes such as BPSK, 16-QAM, DBPSK, and 16-FSK, allowing for a comprehensive assessment of system performance under various modulation strategies. The solid lines depict the exact analytical solutions, while the dashed lines represent the high-SNR asymptotic approximations. Additionally, numerical integration results (circle markers) and Monte Carlo simulations (cross markers) validate the theoretical expressions. The plot shows that coherent modulations consistently outperform non-coherent modulations with the same constellation size, with BPSK and 16-QAM achieving lower error rates than DBPSK and 16-FSK, respectively, at the same SNR levels. Increasing α results in a steeper decline in ABER, confirming its role in mitigating severe fading. The performance gap between coherent and non-coherent schemes widens at higher SNRs, emphasizing the benefits of coherent detection in hyper-Rayleigh conditions. The asymptotic approximations provide reliable error rate estimates beyond $\bar{\gamma }=25$ dB for small α (i.e., α≈1) and for large α beyond 10 dB, reducing computational complexity while maintaining accuracy. The results indicate that selecting an appropriate α value and modulation scheme is crucial for optimizing communication link quality in WSN operating under Fluctuating Nakagami-*m* fading conditions.

**Figure 8:**
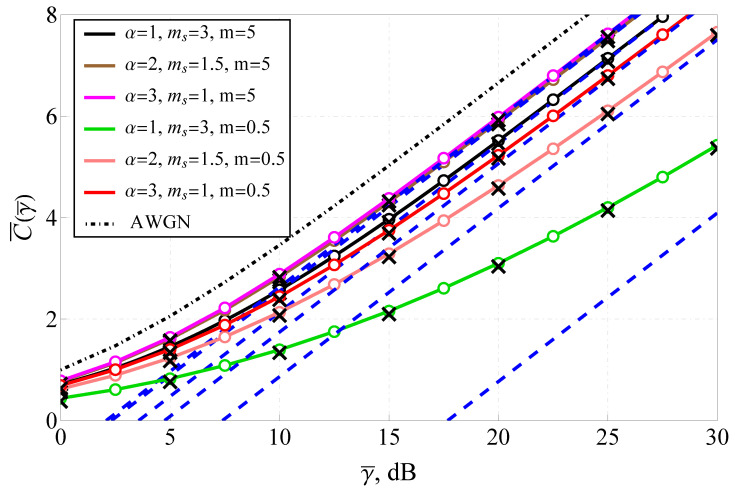
Capacity for various $\bar{\gamma }$ and fading conditions: solid lines—the derived analytic solution (32), dashed blue lines—the derived asymptotic expression (34), circle markers—numeric integration (31), black cross markers—Monte Carlo simulation, black dot-dashed line—AWGN channel.

Figure 8 depicts the ergodic capacity as a function of SNR for different α values illustrating the impact of fading severity on spectral efficiency. The capacity results are compared with the theoretical upper bound for an additive white Gaussian noise (AWGN) channel, represented by a dot-dashed black line. The solid lines correspond to the exact analytical capacity expressions, while the dashed lines depict the high-SNR asymptotic approximations. The figure shows that higher α values yield greater capacity, confirming that less severe fading allows for improved spectral efficiency. At γ=10 dB, the capacity for α=1 is approximately 1.5 bits/s/Hz, whereas for α=3, it exceeds 3 bits/s/Hz, demonstrating a twofold increase in achievable data rates. The capacity curves exhibit logarithmic growth with SNR, aligning with theoretical predictions for fading channels. However, at low α values, the capacity saturates even at high SNRs, indicating that deep fades significantly degrade achievable rates. The Monte Carlo simulation results (black cross markers) and numerical integration results closely follow the analytical predictions, validating the derived expressions. The figure highlights that the α-Fluctuating Nakagami-*m* model effectively captures the impact of fading on channel capacity, providing a useful framework for optimizing transmission strategies.

A cross-comparison between the results validates the statistical accuracy of the model in capturing real-world fading dynamics, reinforcing the reliability of the theoretical performance metrics, and reveals a strong correlation between outage probability and ABER, as both metrics improve significantly with increasing α. This consistency indicates that optimizing α leads to systematic improvements in all key performance indicators (enhancing both link reliability and quality), demonstrating the importance of parameter selection in adaptive wireless communication systems.

### 5.3. Numerical Computation Discussion

Comparing the derived results with those for the underlying Fluctuating Nakagami-*m* model, it is clear that the computational complexity is of the same order. Specifically, both models yield results in terms of either Meijer G-functions or Fox H-functions. Moreover, the expressions for ABER and capacity are provided in terms of a single univariate Fox H-function. In contrast, the classical approach for α-variate fading channels typically relies on Diophantine approximation of α, resulting in ABER and capacity expressions represented as series of Meijer G-functions (see, for example, [40,51]). The number of summands in such series is determined by the approximation, which can be quite large for non-integer α, thereby significantly increasing the overall computational complexity.

Moreover, results for related models (see, e.g., [29,37,38]) are given in terms of multivariate hypergeometric-type functions (such as Meijer G-functions, Fox H-functions, or Kampé de Fériet functions), which are considerably more complex. While univariate hypergeometric-type functions are implemented in most modern Computer Algebra Systems (CASs), their multivariate counterparts are generally not. However, some numerical routines exist for evaluating such functions (see, e.g., [52,53,54,55]).

It must also be noted that even existing CAS implementations of univariate functions present certain issues—such as significant computational slowdown for non-integer α, or divergence for large values of α. To address these drawbacks, as noted in the remarks following the proof of Theorem 1, the derived results can be implemented using univariate contour integrals in the complex domain (see, for example, expression (14) for the MGF). The remaining question is whether this implementation is computationally more efficient than brute-force numerical integration. For further analysis, one restricts attention to ABER calculation for coherent modulation, specifically QPSK.

To this end, ABER will be evaluated in two ways: using numerical integration of (22) (denoted as ${\bar{P}}^{num}er$), and using the derived closed-form expression (24), represented via a Mellin–Barnes integral (denoted as ${\bar{P}}^{cf}er$). Both integrals were computed numerically using Wolfram Mathematica. In the latter case, the integration contour L was chosen as $L:\left(-\frac{\alpha m}{4}-i\delta ;-\frac{\alpha m}{4}+i\delta \right)$ (see the explanation following Theorem 1 for justification), where δ∈R was adjusted to ensure at least 5-digit accuracy while accelerating the computation. The computation times for these two approaches are denoted as $t_{num}$ and $t_{cf}$, respectively.

Thus, the following numerical performance metrics can be defined:

- $\epsilon_{er}=\left|\frac{{\bar{P}}_{er}^{num}-{\bar{P}}_{er}^{cf}}{{\bar{P}}_{er}^{num}}\right|$—relative error of the obtained solution for the ABER;
- $\epsilon_{t}=\frac{t_{num}}{t_{cf}}$—computational time gain/loss of the proposed solution relative to numerical evaluation.

A solution is considered computationally efficient if $\epsilon_{t}>1$ while $\epsilon_{er}<\bar{\epsilon }$, where $\bar{\epsilon }$ denotes the required accuracy threshold.

**Figure 9:**
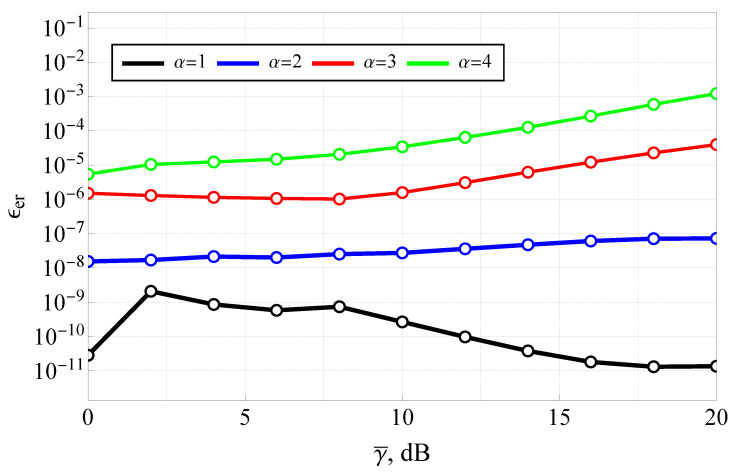
Relative error $\epsilon_{er}$ of the presented solution (relative to the numerical integration) as a function of average SNR.

**Figure 10:**
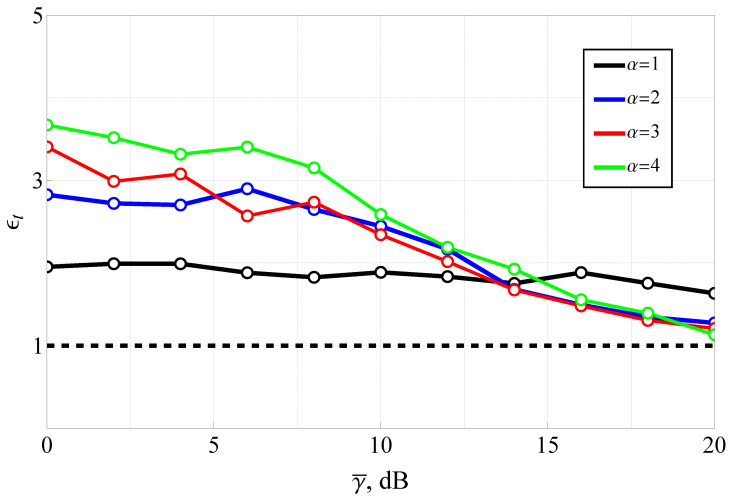
Computation time gain $\epsilon_{t}$ of the presented solution (relative to the numerical integration) as a function of average SNR. The black dashed line denotes equal computational time, i.e., no time gain.

The results for various values of α (with m=2 and $m_{s}=3$) are presented in Figure 9 and Figure 10. It should be noted that the average SNR range was upper-bounded (at 20 dB), since in the high-SNR regime, a highly efficient approximation (derived in Corollary 3) becomes applicable. This approximation offers dramatically lower computational complexity while maintaining excellent accuracy.

The results clearly demonstrate the high accuracy of the closed-form solution across the low-to-moderate SNR range. For instance, at $\bar{\gamma }=10$ dB, the relative error is approximately $5\cdot 10^{-8}$ for α=2, and it remains below $10^{-4}$ even for α=4, confirming the numerical stability and precision of the proposed formulation. At very low SNRs (e.g., $\bar{\gamma }<5$ dB), $\epsilon_{\mathrm{er}}$ achieves its minimum values for α>1 and maximum for α<1, which are still well below $10^{-5}$, which is excellent for most practical purposes. These results verify that the closed-form ABER expression maintains computational integrity for a wide range of α values, and highlight the reliability of the proposed implementation even under complex fading and shadowing conditions. The figure confirms that, despite the involvement of special functions like the Fox H-function, the analytical framework is not only theoretically sound but also numerically feasible for engineering applications.

The results for the computational time gain demonstrate that the analytical solution offers considerable computational efficiency, especially for higher values of α and low-to-moderate SNRs. This efficiency is particularly valuable in scenarios involving large-scale simulations or real-time system optimization, where computational overhead can be a limiting factor. The figure also supports the practical observation that high-SNR asymptotic expressions (such as those in Corollary 3) can be employed beyond $\bar{\gamma }\approx 20$ dB with negligible loss of accuracy, further improving overall speed. Thus, the results validate the efficiency of the proposed Fox H-function-based evaluation framework not only in terms of accuracy (as shown in Figure 9) but also in terms of reduced computational complexity, affirming its suitability for practical deployment in adaptive modulation, link-layer design, and performance analysis in wireless communication systems operating under generalized fading conditions.

## 6. Conclusions

This research introduces the α-Fluctuating Nakagami-*m* (α-FN) fading model as a robust extension of the baseline Fluctuating Nakagami-*m* model, addressing the limitations of conventional fading models in wireless communication analysis. The proposed model incorporates a nonlinear envelope transformation parameter α, which enhances statistical flexibility and improves the fit to empirical fading scenarios. The study provides a complete first-order statistical characterization of the α-FN model, including closed-form expressions for the PDF, CDF, MGF, and raw moments, all with manageable computational complexity. The results demonstrate strong agreement with both numerical integration and Monte Carlo simulations, confirming the analytical derivations’ accuracy. A comparative performance analysis reveals that increasing α significantly reduces outage probability and average bit error rate (ABER), with improvements of several orders of magnitude observed at moderate SNRs. For instance, at $\bar{\gamma }=25$ dB, increasing α from 1 to 4 lowers the outage probability from $10^{-1}$ to $10^{-4}$, reflecting greater link reliability. Similarly, ABER performance for BPSK and 16-QAM improves notably with α, and coherent modulation consistently outperforms non-coherent schemes. The ergodic capacity also scales positively with α, with α=3 yielding twice the capacity of α=1 at γ=10 dB. These trends confirm that α serves as an effective control parameter for optimizing system performance under diverse propagation conditions. The model’s high-SNR asymptotics simplify system evaluation and provide reliable upper bounds on performance. Experimental validation with D2D data shows that the α-FN model outperforms existing models such as IPL and F-Nak in capturing deep fading effects, with the lowest MSE. This flexibility is essential for wireless sensor networks (WSNs), where nodes often operate under harsh and unpredictable fading. The model’s closed-form expressions enable rapid analysis of critical metrics, facilitating real-time adaptation in resource-constrained WSNs. Applications in D2D, mmWave, and THz communications further demonstrate its relevance to emerging technologies. The joint evaluation of fading, shadowing, and nonlinear transformation establishes the α-FN model as a comprehensive statistical tool. Overall, the α-Fluctuating Nakagami-*m* model offers a powerful, empirically validated framework for modeling complex wireless channels and optimizing network performance.

## Data Availability

The data that support the findings of this study are available from the corresponding author upon reasonable request.
